# Adult Anopheles Mosquito Distribution at a Low and High Malaria Transmission Site in Tanzania

**DOI:** 10.1155/2022/6098536

**Published:** 2022-01-10

**Authors:** David Zadock Munisi, Mary Mathew Mathania

**Affiliations:** ^1^Department of Microbiology and Parasitology, School of Medicine and Dentistry, The University of Dodoma, P.O. Box 259, Dodoma, Tanzania; ^2^Department of Basic and Behavioral Sciences, School of Nursing, Saint John's University of Tanzania, Dodoma, Tanzania

## Abstract

Malaria parasites are only transmitted by female mosquitoes of the genus Anopheles; hence, the disease's distribution is linked to that of the vector mosquitoes. As such, the goal of this study was to find out the spatial and temporal distribution of Anopheles mosquito adults in the research sites. This was a repeated cross-sectional ecological study that took place in Morogoro and Dodoma, Tanzania. Vacuum aspiration was used to collect mosquitoes both outside and inside human dwellings. All mosquito-related data was collected and entered into appropriate data collection forms. Female mosquitoes were recognized morphologically using Gillies and Coetzee morphological criteria, followed by PCR. In total, about 2742 *Anopheles* mosquitoes with an average collection of 18.21 ± 1.12 per day were collected outside human houses of which 1717 (10.51 ± 1.17) and 1025 (8.42 ± 1.41) were collected from Morogoro and Dodoma, respectively. Of the captured mosquitoes, 89.0%, 10.0%, and 1.0% were recognized as *Anopheles arabiensis*, *Anopheles gambiae s.s.*, and *Anopheles quadrianulatus*, respectively. The distribution varied significantly with seasons, whereby 302 (4.72 ± 1.04) and 2440 (12.96 ± 1.52) mosquitoes were captured in the cold-dry and warm-wet season, respectively (*p* < 0.0001). Of the captured mosquitoes, 42.33%, 16.33%, 14.96%, and 4.27 were found on the ceiling, stored junks, verandas, and barks/tree, respectively. In malaria-endemic countries, vector control forms an important component of the malaria control efforts. This study found significant variation of Anopheles mosquito abundance in time and space with *Anopheles arabiensis* being the most predominant malaria vector. This signifies the need to introduce mosquito control methods that will target the less anthropophilic *Anopheles arabiensis* or the immature aquatic stages. The study further found that underbeds, store room/piled bags, and undisturbed curtains were the most preferred resting places by mosquitoes signifying to be the most effective strategic sites for spraying insecticides during the implementation of indoor residual spraying (IRS).

## 1. Introduction

Malaria is a major public health issue in Tanzania, with 93 percent of the population being at risk, the disease is the main national cause of outpatient visits and admissions in health care facilities, particularly among children under the age of five and pregnant women [1]. The disease is caused by protozoan parasites of the genus Plasmodium which are exclusively transmitted by female Anopheles mosquitoes [2]; consequently, the distribution of the disease correlates with the distribution of the vector mosquitoes [3].

Macro (temperature and topography) and micro (availability of aquatic biological niches) factors influence the occurrence of vector Anopheles mosquitoes. Within a district, region, country, or continent, climate and topography are significant factors of macro spatial-temporal Anopheles mosquito distribution [3–6]. The availability of aquatic habitats close to human dwellings determines the micro -spatial occurrence of vector mosquitoes within the community [7, 8]. Adult mosquito distributions are typically seasonal and follow rainfall patterns, resulting in population densities differing between and within countries [9]. The knowledge of malaria transmission dynamics, particularly the temporal and spatial distribution of Anopheles mosquito vector populations, is generally low due to Tanzania's large geographic size and different ecoclimatic conditions. It is widely acknowledged that vector control plays a critical role in achieving malaria elimination in endemic areas [10, 11]. Understanding the temporal and spatial distribution of sympatric malaria vector species is important for effective vector control in malaria-endemic countries like Tanzania [2, 12]. This research intended, therefore, to investigate distribution in time and space of Anopheles mosquito adults so as to have a better understanding of the distribution of these important malaria vectors to be used in implementing different vector control measures that target adult mosquitoes.

## 2. Methodology

### 2.1. Study Area

The research was carried out in Tanzania's Dodoma and Morogoro regions. Morogoro has a high malaria prevalence of 9.5 percent, while Dodoma has a low malaria prevalence of 0.6 percent [13]. The Morogoro municipality is located in Tanzania's eastern region, about 169 kilometers west of Dar-es-Salaam and about 223 kilometers east of Dodoma, the country's capital. Short rains fall in December and January, and long rains fall from March through June. During the rainy seasons (December–May), the mean annual rainfall is about 783.5 mm, with a mean relative humidity of about 72 percent, a lowest temperature of 22°C, and a high temperature of 33°C during the wet season. The minimum and maximum temperatures throughout the cold season (June-September) are 15°C and 19°C, respectively. Dodoma is 486 kilometers west of Dar-es-Salaam, located at 6°25′S and 35°75′E. The study areas have been described in details in our earlier publication [14].

### 2.2. Study Design

The research was a repeated cross-sectional ecological study. The data was gathered throughout two seasons: the cold-dry season from June to September 2014 and the hot-wet season from January to February 2015. Adult mosquitoes were collected in the same areas inside and outside human houses during both seasons of the study.

### 2.3. Sampling Techniques

Purposefully, two wards were chose both in Dodoma and Morogoro urban on the basis of their having seasonal and temporary breeding habitats (presence of artificial breeding sites, natural wetlands, and rice fields) as well as a local high incidence of malaria [15, 16].

### 2.4. Data Collection Procedure

#### 2.4.1. Data Collection on Adult Mosquito

From the sampled wards, ten houses were randomly selected from each street in Morogoro and Dodoma. A handheld global positioning system (GPS) device was used to map out the homes that were included in the mosquito sampling. Mosquitoes were collected using a vacuum backpack aspirator outside and inside human dwellings in places like verandas, unfinished buildings (inside and out), fences, stored junks, and tree holes, as well as behind curtains, underbeds, and under couches, while knockdown catches were done in places like storerooms with pyrethrum aerosol spray (from Rungu®-aerosol spray cans). The catching took place in the mornings between 0700 and 1000 hrs and in the evenings between 1700 and 1900 hrs. All mosquito-related data was filled into the appropriate data collection forms. [17]. A vacuum aspirator with a collection cup at the back was used to gather mosquitoes trapped in the field during mosquito collection. The mosquitoes in the collecting cup were emptied into transportation plastic cups (labeled with street names) and moved to the field before being counted and sorted at the insectary. The morphological keys of Gillies and Coetzee were used to identify female mosquitoes. Following morphological identification, mosquitoes were analyzed to species siblings by PCR.

#### 2.4.2. Differentiation of Anopheles Species by Polymerase Chain Reaction Analysis

PCR was used to identify a total of 100 mosquitoes that were randomly selected from a total of 2742 which were morphologically identified to be adult female Anopheles mosquitoes. All of the female Anopheles that were chosen were taken to the lab for DNA extraction and PCR analysis. The DNA extraction protocol is as described in our earlier publication [14]. The isolated DNA pellet was kept at -20°C until it was used to conduct the PCR analysis.

PCR techniques developed by Scott et al. [18] were used to differentiate the *Anopheles gambiae* complex. The PCR amplification for the *Anopheles gambiae* complex was carried out using universal and species-specific primers, as described in our previous publication [14]. The species-specific nucleotide sequences in the ribosomal DNA intergenic spacer (IGS) were used to identify the An. gambiae complex using three deferentially sized amplicons. Anopheles gambiae s.s. (390 bp), Anopheles arabiensis (315 bp), and Anopheles quadriannulaus (150 bp) were the species extracted product sizes [18]. A UV transilluminator was used to visualize the amplified DNA on a 2.0 percent agarose gel stained with ethidium bromide. The primers for the Anopheles gambiae complex are listed below [14]:

*Primers*

*UN*: 5′-GTG TGC CCC TTC CTC GAT GT-3′

*GA*: 5′-CTG GTT TGG TCG GCA CGT TT-3′

*AR*: 5′-AAG TGT CCT TCT CCA TCC TA-3′

*QD*: 5′-CAG ACC AAG ATG GTT AGT AT-3′

#### 2.4.3. Establishment of Sheltering Places of Vector Anopheles Mosquitoes during Dry-Cold Season (June-September)

From June to September 2015, researchers conducted an identification of mosquitoes hiding places in randomly selected houses in each study site (ten houses each in Morogoro and Dodoma). Mosquitoes were collected using spray sheet collection method with knockdown catches using pyrethrum aerosol spray (from Rungu®-aerosol spray cans) in places like storerooms, while an aspirator was used to collect mosquitoes in undisturbed places like under the bed, store rooms, behind the curtains, shelves, and ceilings. Following that, all mosquitoes caught in aspirator cups and knockdown catches (fell on the white sheet) were temporarily placed in labelled carrying plastic cups before being counted; they were then sorted by sex and identified morphologically in the laboratory. Each female Anopheles mosquito was maintained in an Eppendorf filled with silica gel after morphological identification. The silica gel was employed to keep mosquitoes dry as they waited to be killed.

### 2.5. Data Processing and Analysis

To investigate the impact of research site variables on Anopheles mosquito distribution, data on the number of vector mosquitoes collected in various locations were modelled using a Poisson regression model. Mosquitoes were collected and taken to the laboratory for morphological and molecular analysis. As indicated in General Laboratory Analysis, identified vector mosquitoes were sampled and submitted for PCR identification.

### 2.6. Ethical Considerations

The Sokoine University of Agriculture's (SUA) Directorate of Research and Postgraduate Studies provided ethical approval for the research. The district and local ward administrations provided permission to conduct the study. After presenting the study's objectives to the head of the household, written consent was acquired to set up the trials in selected households to evaluate the Umbrella-topped Contaminating Device (UtMCD).

## 3. Results

### 3.1. Adult Anopheles Mosquito Distribution Based on Study Characteristics

Outside human residences, a total of 2742 Anopheles mosquitoes were captured, with an average of 18.21 ± 1.12 per day, with 1717 (10.51 ± 1.17) being collected in Morogoro. Only 302 (4.72 ± 1.04) were captured during the dry-cold season (June-September), while 2440 (12.96 ± 1.52) were collected during the wet-hot season (March-May). The quantity of collected mosquitoes from different sites was analyzed, and Mahita in Morogoro (621 (21.64 ± 2.74)) and Swaswa in Dodoma (576 (18.87 ± 2.59)) had the largest quantities of mosquitoes collected (Table 1).

### 3.2. Adult Anopheles Mosquito Distribution in Different Sites

**Figure 1:**
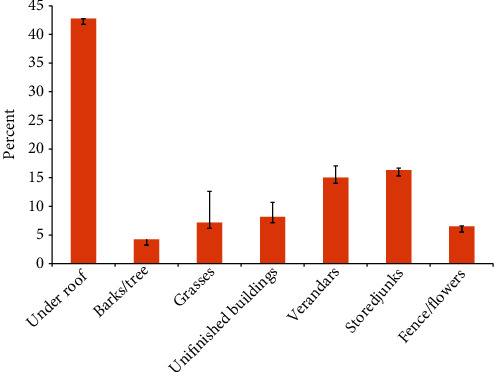
Adult *Anopheles* mosquito percentage distribution by location. Standard error of the mean is represented by error bars.

**Figure 2:**
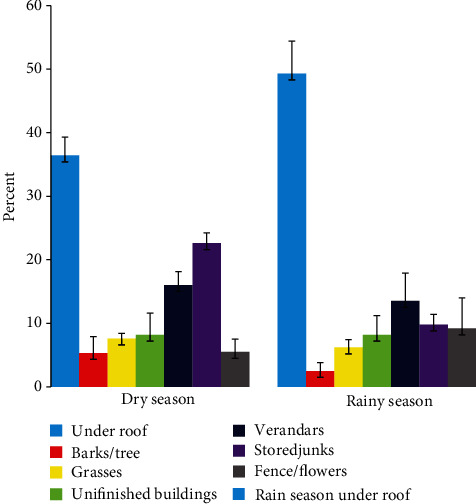
Adult mosquito distribution by season and place as a percentage. The standard error of the mean is represented by the error bars.

Out of 2742 of vector *Anopheles* mosquitoes caught, majority (42.33%) were found on the ceiling, followed by 16.33%, 14.96%, and 4.27% in stored junks, verandas, and barks/tree, respectively (Figure 1). The variation in the distribution of vector mosquitoes by location was observed to be statistically significant with respect to season (*χ*^2^ = 168.79, *p* = <0.0001), whereby in dry-cold season (June-September), a total of 320 vector *Anopheles* mosquitoes were caught outside houses of which 36.47%, 22.43%, 16.11%, and 8.55% were collected from ceiling, stored junks, verandas, and inside unfinished buildings, respectively. In wet-warm season (March-May), out of 2440 *Anopheles* mosquitoes caught, the highest percent (49.03%) was found on ceiling followed by 13.64%, 9.35%, and 9.28% in verandas, stored junks, and fence/flower, respectively (Figure 2). To determine the effect of season on the distribution of the collected adult mosquitoes, a Poisson regression model was constructed. The model's results, which are presented in Table 2, show that after adjusting for the study area, the distribution of adult mosquitoes was significantly associated with season (*p* < 0.0001), with the mean number of mosquitoes collected during the cold-dry season (June-September) being nearly twice as much to that collected during the rainy season (March-May) (AMR = 1.6, *p* < 0.0001). Furthermore, the average number of adult mosquitoes collected in Morogoro (AMR = 1.34, *p* < 0.0001) was significantly higher than that in Dodoma (AMR = 1.34, *p* < 0.0001) (Table 2).

### 3.3. The Species Composition of Adult Mosquitoes Collected

PCR was used to identify 100 mosquitoes randomly picked from a total of 2742 morphologically identified adult female Anopheles mosquitoes. Anopheles arabiensis accounted for 89.0% of the total, whereas Anopheles gambiae s.s. accounted for 10.0%, and Anopheles quadrianulatus accounted for only1.0%.

### 3.4. Anopheles Mosquitoes' Sheltering Places during Cold-Dry Seasons

During the cold-dry season (June–September), 1541 mosquitoes were collected inside human dwellings, with an average daily collection of 19.26 ± 1.81. Mosquitoes have been found to take up residence in various locations within homes. According to the data, Dodoma had the highest mean number of collected mosquitoes from sheltering locations (26.63 ± 2.37), with Swaswa street having the highest mean Anopheles catches (32.07 ± 3.33) (Table 3).

### 3.5. Anopheles Mosquito's Percentage Distribution according to Sheltering Places

**Figure 3:**
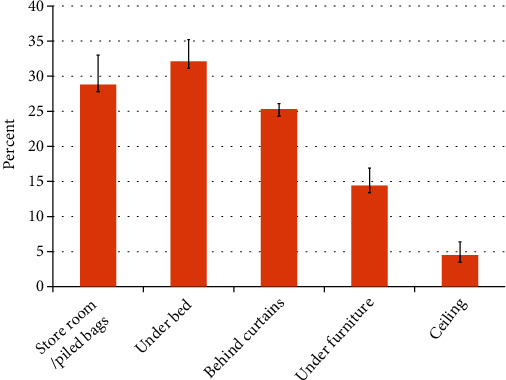
*Anopheles* mosquitoes' percentage distribution according to sheltering places inside human houses. Error bars indicate standard error (*n* = 1541).

Of all the collected mosquitoes (1541), the majority (32.06%) were collected underbeds, followed by 25.31%, 23.75% and 4.48% behind undisturbed curtains, store room/piled bags, and ceilings, respectively (Figure 3).

### 3.6. Poisson Regression Model for the Effect of Region on Mosquito Distribution

The Poisson regression model (Table 4) for the effect of region on the distribution of Anopheles mosquitoes during the sheltering period in dry season revealed that the average number of vector Anopheles mosquitoes captured in Morogoro (MR = 0.56, *p* = 0.0002) was significantly lower than that captured in Dodoma.

## 4. Discussion

Vector control forms an important part of malaria control program. Effective vector control is dependent on a good understanding of the distribution of the vector mosquitoes in endemic communities [2, 10–12]. We report the distribution of vector Anopheles mosquito as determined in the two study areas.

Significant variation on adult mosquito abundance between places has been reported in other places, as a possible explanations, availability of favourable larval habitats, the distance from residents or houses to larval habitats, and the types of house roofs have been postulated [19]. Similarly, our study reports not only a relatively higher numbers of mosquitoes in the region known to have higher malaria prevalence, i.e., Morogoro as compared to Dodoma, but also expectedly, mosquitoes were plentiful during rainy season than during cold/dry season owing to the abundancy of vector larval breeding sites in wet/hot season than in the other season. The observed variation of mosquito abundance in time was mainly as a result of rainfall changes in different seasons. Anopheles mosquito abundance may be used as a proxy of malaria transmission intensity and therefore used to guide focused malaria control interventions in endemic areas as countries move towards malaria focal elimination. As already known, the distribution of malaria can be quite variable even over very short distances within the same village; in our study, this was evident as we found high mosquito abundance in Mahita and Swaswa in Morogoro and Dodoma, respectively, as compared to other parts. Knowledge of this spatial distribution of Anopheles mosquito vector dictated by certain environmental, climatic, and anthropogenic factors can be deployed when defining residual malaria transmission while advancing to malaria elimination.

*Anopheles arabiensis* was the main malaria vector in the research area during both seasons. The use of LLINs/ITNs and IRS, which reduce the extremely anthropophilic Anopheles species *Anopheles gambiae s.s.*, which is now being replaced by *Anopheles arabiensis*, is most likely the reason for the increasing abundance of Anopheles arabiensis [12]. In different places in Tanzania, it has been observed that there is a significant shift in the Anopheles mosquito abundance from a predominantly *Anopheles gambiae s.s.* to *Anopheles arabiensis* [12, 20–22]. This has also been observed in the study area especially Morogoro where in previous years, *Anopheles gambiae s.s.* were the most wide spread species but as observed in our study, the surveyed wards had over 80% of collected *Anopheles* being *Anopheles arabiensis* [20, 21].

Expectedly, during the cold-dry season, vector mosquitoes disappear to aestivate/hibernate [23–25]. This study has found that the majority of mosquitoes hide inside buildings during the dry/cold season in the dark and/or regions with little or no disturbances, whereby underbeds, behind undisturbed curtains, store rooms/stored junks, and under furniture were the most commonly preferred hiding places during the dry/cold season. Merdic and colleagues discovered that during the cold/dry season, vector Anopheles mosquitoes concealed in cellars, deep basements, dark rooms with small windows, and on dirt floors formed of bricks [26]. According to many studies that explain how mosquitoes become immobile during the cold/dry season [23–25, 27], spraying methods that knock down mosquitoes can be used during the cold season to kill many mosquitoes, reducing population density in the community before the warm/wet seasons when mosquitoes emerge from hiding places. However, in Tanzania, indoor residual spraying (IRS) is a malaria management approach that targets adult mosquito vectors [28]. A good understanding of the resting behavior of local mosquitoes is required for an efficient indoor residual spraying (IRS) campaign. According to the findings of our study, spraying underbeds, storage rooms/piled bags, and undisturbed curtains is likely to accomplish greatest mosquito control, as these are mosquitoes' preferred resting spots.

## 5. Conclusion

Vector control is an important component of malaria control in endemic countries. A good understanding of the spatial-temporal distribution of the Anopheles mosquito vector forms a necessary prerequisite in developing a sound and effective vector control program. This study found significant variation of Anopheles mosquito population in time and space with *Anopheles arabiensis* being the most predominant malaria vector found. The predominance of *Anopheles arabiensis* which is a zooanthropohilic mosquito as compared to the highly anthropohilic *Anopheles gambiae* signifies success of LLINs/ITNs and IRS. However, this also signifies the need to introduce methods in malaria control arsenal of methods that will also kill the less anthropophilic *Anopheles arabiensis* or methods directed towards the immature aquatic stages which will indiscriminately kill all Anopheles larva. The study further found that underbeds, store room/piled bags, and undisturbed curtains were the most preferred resting places by mosquitoes signifying to be the most effective strategic sites for spraying insecticides during the implementation of IRS.

## Figures and Tables

**Table 1:** Adult Anopheles mosquito distribution based on study characteristics.

Variable	Number	Mean ± SE
Region		
Morogoro	1717	10.51 ± 1.17
Dodoma	1025	8.42 ± 1.41

Season		
Dry	302	4.72 ± 1.04
Wet	2440	12.96 ± 1.52

Area		
Morogoro		
Tupendane	272	7.20 ± 1.54
Visegese	164	8.37 ± 1.47
Mahita	621	21.64 ± 2.74
Mwembeni	447	13.55 ± 2.15
Misufini	213	11.65 ± 2.07
Dodoma		
Mnarani	449	14.97 ± 2.34
Swaswa	576	18.87 ± 2.59

**Table 2:** The Poisson regression model's parameter estimates, adjusted mean ratio (AMR), and significance levels for the effect of region and season on adult mosquito distribution.

Parameter	Estimate ($\hat{\beta }$)	SE	*p* value	AMR
Intercept	2.470	0.040	<0.0001	11.85
Region				
Morogoro	0.290	0.040	<0.0069	1.34
Dodoma	1			
Season				
Dry	0.470	0.030	<0.0001	1.60
Rain	1			

**Table 3:** *Anopheles* mosquito sheltering in different sites in Dodoma and Morogoro, mean distribution.

Variable	Mean ± SE
Region	
Morogoro	14.84 ± 1.03
Dodoma	26.63 ± 2.37
Study area (streets)	
Morogoro	
Mwembeni	12.80 ± 1.81
Visegese	12.40 ± 1.52
Tupendane	14.60 ± 1.93
Misufini	15.40 ± 1.98
Mahita	17.00 ± 2.39
Dodoma	
Mnalani	21.20 ± 2.08
Swaswa	32.07 ± 3.33

**Table 4:** Poisson regression model's parameter estimates and adjusted mean ratio (MR) for the effect of region on distribution of sheltering *Anopheles* mosquitoes collected during dry-cold season.

Parameter	Estimate ($\hat{\beta }$)	SE	*p* value	MR
Intercept	3.280	0.110	<0.0001	26.630
Region				
Morogoro	-0.580	0.160	0.0002	0.560
Dodoma	1			

## Data Availability

Data underlying this study are available upon request.
